# Biological characterization and *in vitro* fungicide screening of a new pathogen of basal stem rot of *Schisandra chinensis* in Jilin Province, China

**DOI:** 10.3389/fmicb.2025.1633730

**Published:** 2025-06-30

**Authors:** Taiping Tian, Mengqi Wang, Jingmeng Zhu, Yining Sun, Mingjie Ma, Guoliang Liu, Jinshuo Wang, Bing Zhao, Yiping Yan, Yue Wang, Yanli Wang, Changyu Li, Dongyin Wang, Fang Yang, Wenpeng Lu, Peilei Xu

**Affiliations:** ^1^CAAS/Jilin Provincial Key Laboratory of Traditional Chinese Medicinal Materials Cultivation and Propagation, Institute of Special Animal and Plant Sciences, Changchun, China; ^2^College of Agriculture, Yanbian University, Yanji, China; ^3^Institute of Science and Technology Information of Jilin Province, Changchun, China; ^4^Jilin Provincial International Cooperation Key Laboratory for Science and Technology Innovation of Special Animal And Plants’, Changchun City, Jilin Province, China

**Keywords:** *Schisandra chinensis*, basal stem rot, *Ilyonectria robusta*, biological characteristics, fungicide screening

## Abstract

**Introduction:**

*Schisandra chinensis* (Turcz.) Baill. is a widely cultivated medicinal plant with significant economic value and serves as a major component of specialty agriculture in Jilin Province, China. In 2024, a novel basal stem rot disease was observed in Latai Village, Liudaogou Town, Linjiang City, Jilin Province, posing a potential threat to local production.

**Methods:**

Field symptoms were characterized by stem-base necrosis, vascular browning, wilting, and plant death, often accompanied by soft rot under high humidity. The causal pathogen was isolated and identified through morphological observation and sequencing of *ITS*, *β*-tubulin (*TUB*), and *TEF1-α* genes. Pathogenicity was confirmed via Koch’s postulates on detached stems, seedlings, whole plants, and tissue-cultured plantlets. Biological characteristics were evaluated under different media, temperatures, light conditions, pH levels, and carbon/nitrogen sources. Additionally, seven fungicides (three chemical and four botanical) were tested for inhibitory activity *in vitro*.

**Results:**

The pathogen was identified as *Ilyonectria robusta*, marking the first global report of this species causing basal stem rot on *S. chinensis*. Optimal growth conditions included CDA or CMA media, 25°C, continuous darkness, and pH 7. Sodium nitrate and lactose were the most favorable nitrogen and carbon sources, respectively. Among the fungicides tested, 98% fluazinam showed the strongest inhibitory effect with an EC₅₀ value of 5.658 mg/L.

**Discussion:**

This study confirms *I. robusta* as a novel and virulent pathogen of *S. chinensis* in Jilin Province. The findings provide foundational data on its biology and pathogenicity and identify fluazinam as a promising candidate for integrated management of basal stem rot disease.

## 1 Introduction

*Schisandra chinensis* (Turcz.) Baill. is a genus of *Schisandra* in the family *Schisandraceae*, commonly known as Xuan and, will and, five plums, habitually known as the northern *schisandra*, dry fruit for medicine (Nowak et al., 2019; Szopa et al., 2017). It possesses various pharmacological activities, including blood glucose-lowering, anti-cancer, liver-protective, anti-inflammatory, and antioxidant effects (Olas, 2023). As one of China’s traditional and precious medicinal materials, it is first recorded in the *Shennong Ben Cao Jing* and is revered as a “top-grade medicine” (Meng et al., 2025). Currently, *S. chinensis* is used in hundreds of traditional Chinese medicine prescriptions and over 100 proprietary Chinese medicines (Yang et al., 2022). In recent years, with the growing value of *Schisandra* extract, the cultivated area has expanded rapidly, leading to an increasing issue of pest and disease outbreaks. Common diseases include leaf spot (Wang et al., 2012), leaf blight (Yan et al., 2023), fruit rot (Yu et al., 2011), root rot (Shen et al., 2022), and basal stem rot, all of which severely impact the yield and quality of *S. chinensis* and greatly hinder the development of the industry.

Among these, *I. robusta*, the pathogen responsible for black foot disease, was first discovered in Brazil in 2014 (Dos Santos et al., 2014) and reported in Spain in 2018 (Martínez-Diz et al., 2018). In 2015, root rot caused by this pathogen was first discovered in Jilin Province, China (Lu et al., 2015), and it was subsequently reported in Sichuan Province and Chongqing City in 2022 and 2023 (Lyu et al., 2023; Zheng et al., 2022). However, there are no published reports on *I. robusta* causing basal stem rot in *S. chinensis*. Field surveys revealed that the early symptoms of *Schisandra* basal stem rot include the formation of brown lesions at the basal stem. As the disease progresses, these lesions expand, and the junctions between the diseased and healthy tissue become rotten, turning deep brown (Lyu et al., 2023; Zheng et al., 2022). In addition to its medicinal value, *S. chinensis* plays a crucial role in local economies, particularly in northeast China, where its cultivation has become a vital source of income for many rural communities. The growing demand for its extracts in both pharmaceutical and functional food industries has driven the rapid expansion of its planting area and increased its economic importance nationwide.

In 2024, during a disease survey in major *Schisandra* cultivation areas in Jilin Province, it was found that this disease was widespread, with field infection rates exceeding 70%. To enable accurate prevention and control of *Schisandra* basal stem rot, this study focused on isolating and purifying the pathogen, assessing its pathogenicity, conducting morphological observations, and molecular biological identification of samples collected from *Schisandra* cultivation areas in Jilin Province. Additionally, the study investigated key biological characteristics of the pathogen—such as temperature, pH, and nutrient preferences—to understand its environmental adaptability, and screened *in vitro* for effective fungicides to identify potential agents for chemical control. This research aims to provide theoretical guidance for the scientific prevention and control of *Schisandra* basal stem rot, contributing to the efficient and stable production of Schisandra in Jilin Province and supporting the sustainable development of the industry.

This study reports for the first time a basal stem rot disease occurring in the cultivation area of *S. chinensis* at Latai Village, Liudougou Town, Linjiang City, Jilin Province, China. In order to clarify the cause of the disease and to provide a scientific basis for future prevention and control in this area, the aim of this paper was to identify the pathogen species, assess their biological responses to key environmental factors, and screen *in vitro* for fungicides with good preventive efficacy.

## 2 Materials and methods

### 2.1 Disease sample survey and collection

In July 2024, samples of typical basal stem rot disease were collected from a cultivation site of *S. chinensis* in Latai Village, Liudougou Town, Linjiang City, Jilin Province, China (latitude 41°40′57″N, longitude 127°2′50″E). According to the typical symptoms exhibited by the diseased plants, such as basal stem rot, browning of vascular bundles and clear junction of diseased and healthy tissues, 12 representative diseased plants from the field were screened as research subjects. The specific operations were as follows:

(1) Record the morphological characteristics of the diseased plants and take photos for storage;
(2) Use sterile scissors to cut the tissue at the base of the diseased stems, and select the areas with clear disease-health junction and obvious browning but not completely decayed, avoiding the sampling of self dry rot or secondary infested areas;
(3) Use a small sterile spatula to gently dig up the root system, removing adherent soil and retaining the stem base and main root;
(4) Place the samples from each plant in pre-cooled sterile sampling bags, number them uniformly, place them in a low-temperature ice box for cold storage, and transport them back to the laboratory within 24 h for pathogen isolation and identification.

### 2.2 Pathogen isolation and purification

The pathogen was isolated from the diseased basal stem tissue of *S. chinensis* using the routine tissue isolation method (Liu et al., 2022). Under sterile conditions, 5 mm × 5 mm tissue blocks were excised from the epidermis, phloem, cambium, xylem, and pith of the basal stem segment using sterilized scissors and a surgical knife. The tissue blocks were then disinfected by immersion in 1% mercuric chloride solution for 2 min, followed by disinfection in 75% ethanol for 1 min, and then washed 4–5 times with sterile water. Afterward, the tissue blocks were placed on sterilized filter paper to absorb excess moisture. Using sterilized forceps, the tissue blocks were gently pressed to adhere to potato dextrose agar (PDA) plates containing 50 μg·mL^−1^ streptomycin sulfate. The plates were sealed and incubated at 25°C in the dark, with regular observations of colony growth. After 3–4 d, typical colonies, free of contaminants, that grew from the isolated tissue blocks were selected. The hyphal edges of the colonies were transferred to fresh PDA plates for continuous purification by subculturing three times. Once a pure culture was obtained, it was transferred to new media and incubated in the dark at 25°C for about 5 days. The morphological characteristics of the colonies and the color of the hyphae were observed and recorded.

### 2.3 Pathogenicity test

#### 2.3.1 Inoculation of basal stem segments

The purified isolates were inoculated onto potato dextrose agar (PDA) plates and incubated at 25°C for 7 days. After the incubation, 8 mm diameter mycelial discs were cut from the colonies. Healthy *S. chinensis* plants were selected, and 8 cm long stem segments were cut. These segments were placed in petri dishes lined with moist sterile filter paper, with the ends of the stems wrapped in sterile, moist cotton to maintain humidity. After making artificial wounds on the surface of the stem segments, the mycelial side of the mycelial discs was placed onto the wounds for inoculation, with a sterile PDA agar block used as the control. Six stem segments were set up for each treatment. The petri dishes were sealed and incubated at 25°C in the dark with humid conditions for 7 days. After incubation, the stem segments were observed and the disease symptoms were recorded.

#### 2.3.2 Inoculation of tissue culture seedlings

The purified isolates were inoculated onto potato dextrose agar (PDA) plates and incubated at 25°C for 7 days. After the incubation, 8 mm diameter mycelial discs were cut and inoculated onto the basal stems of 30-day-old healthy *S. chinensis* tissue culture seedlings, with one mycelial disc per seedling. A total of 12 seedlings were inoculated. After inoculation, the tissue culture seedlings were placed in a growth chamber set to 25°C and 70% relative humidity. A sterile PDA agar block was used as the control. After 10 days of incubation, disease symptoms were observed and the incidence of the disease was recorded for each treatment.

#### 2.3.3 Inoculation of detached seedlings

Purified isolates were inoculated onto potato dextrose agar (PDA) plates and incubated at 25°C for 7 days. After incubation, 8 mm-diameter mycelial plugs were excised from colony margins using a sterile cork borer. Ten healthy, 20-day-old *S. chinensis* seedlings were selected: five seedlings received mycelial plugs as the treatment group, and five received sterile PDA plugs of the same size as the control group. All seedlings were placed in Petri dishes lined with moist sterile filter paper, with their roots wrapped in sterile, moistened cotton to maintain high humidity. After inoculation, dishes were sealed with transparent tape and incubated in a completely dark growth chamber at 25°C and 70% relative humidity for 10 days. Disease symptoms were observed and recorded at the end of the incubation period.

#### 2.3.4 Inoculation of potted seedlings

##### 2.3.4.1 Preparation of potted seedlings

*Schisandra* seeds, treated with sterilization, were sown in a sand-stone mixed substrate and subjected to a temperature accumulation method to promote germination at room temperature. As the seeds absorbed water and expanded, the seed coat gradually swelled and small buds began to form. Once germination was clearly observed, the seeds were carefully transferred to large seedling trays to allow for growth in a spacious environment. When the seedlings reached a height of approximately 5 cm, they were transferred to small nutrient pots (8 cm × 8 cm in size) for more refined management to obtain standard potted seedlings for subsequent pathogenicity testing.

##### 2.3.4.2 Inoculation and pathogenicity test

The purified isolates were inoculated onto potato dextrose agar (PDA) plates and incubated at 25°C for 7 to 12 days to allow sufficient growth and sporulation. After incubation, the spores were scraped from the surface of the culture medium and suspended in sterile distilled water to prepare a spore suspension, which was then filtered through sterile gauze to remove mycelial fragments. The spore concentration was adjusted to 1 × 10^6^ spores/mL using a hemocytometer (Li et al., 2024). Healthy 55-day-old potted *S. chinensis* plants were selected, and after lightly injuring the basal stem, 100 μL of the spore suspension was applied to the wound site for inoculation. One inoculation was applied per plant, with a total of 6 plants inoculated. After inoculation, the wound area was wrapped with sterile, moist cotton and sealed with a plastic bag to maintain humidity for 24 h. The plants were then placed in an incubator set to 25°C and 70% relative humidity. Plants treated with sterile water served as the control. After 14 days of incubation, disease symptoms were observed and recorded for each treatment.

### 2.4 Identification of the pathogen

#### 2.4.1 Morphological characterization

A sterile 8 mm diameter cork borer was used to sample the margin of purified pathogenic colonies, which were then transferred onto potato dextrose agar (PDA) plates and incubated at 25°C for 7 days. During the incubation period, the colony morphology was recorded and observed. The hyphal characteristics of the pathogen were examined and described under a light microscope. After 15 days of incubation, hyphal samples were collected and placed on a glass slide, followed by the addition of 100 μL of sterile water. The conidiophores and conidia of the pathogen were further observed under a light microscope, and their morphology, pigmentation, and dimensions were described.

#### 2.4.2 Molecular characterization

Genomic DNA of the pathogen cultured for 7 days was extracted using the Ezup Column Fungal Genomic DNA Extraction Kit. Specific primers targeting the internal transcribed spacer (ITS) region, the translation elongation factor 1-alpha gene (*TEF1-α*), and the *β*-tubulin gene (*TUB2*) were used for gene fragment amplification (primer information is listed in Table 1). The total volume of the PCR amplification reaction was 25 μL, containing 12.5 μL of 10 × PCR Buffer, 1 μL of forward primer (10 μM), 1 μL of reverse primer (10 μM), 1 μL of template DNA, and 9.5 μL of ddH₂O. The PCR program was set as follows: initial denaturation at 95°C for 5 min; followed by 30 cycles of denaturation at 94°C for 30 s, annealing at 56–57°C for 30 s (specific annealing temperatures are shown in Table 1), and extension at 72°C for 90 s; with a final extension at 72°C for 10 min. PCR products were detected by electrophoresis on 1% agarose gels and then subjected to bidirectional sequencing at Shanghai Sangon Biotech Co., Ltd. The obtained sequences were compared using BLAST searches in the NCBI database, and homologous reference sequences were downloaded. The sequencing data were submitted to the GenBank database to obtain accession numbers. Sequence alignment and trimming were performed using MEGA version 11.0, and a phylogenetic tree was constructed based on the Maximum Likelihood (ML) method with 1,000 bootstrap replicates. The pathogen species were ultimately identified by combining phylogenetic analysis with colony morphological characteristics.

**Table 1:** Primer information for amplification of *I. robusta* gene sequences.

Gene region	Primer pair (5′ → 3′)	Annealing temperature (°C)	References
ITS	ITS1: TCCGTAGGTGAACCTGCGG ITS4: TCCTCCGCTTATTGATATGC	56	White et al. (1990)
*TEF1-α*	EF1: ATGGGTAAGGAGGACAAGAC EF2: GGARGTACCAGTSATCATGTT	56	O’Donnell et al. (1998)
*β-Tubulin*	BT3: CCCGTATGGCTTCACCGAGC BT4: CTGAACCGAGGAGGAGGTGT	57	O'Donnell and Cigelnik (1997)

### 2.5 Biological characterization of the pathogen

#### 2.5.1 Effects of different nutrient media on mycelial growth of *Ilyonectria robusta*

An 8 mm mycelial plug was excised from the margin of purified colonies using a sterile cork borer and inoculated onto eight different media: potato dextrose agar (PDA), cornmeal agar (CMA), Czapek-Dox agar (CDA), carrot agar (PCA), oatmeal agar (OA), malt extract agar (MEA), potato sucrose agar (PSA), and water agar (WA). Four replicates were set for each treatment. All plates were incubated at 25°C in darkness for 7 days. At the end of the incubation period, the colony diameter was measured using the cross-measurement method (Usman et al., 2022).

#### 2.5.2 Effects of different carbon and nitrogen sources on mycelial growth of *Ilyonectria robusta*

Czapek-Dox agar (CDA) was used as the base medium, with sodium nitrate as the fixed nitrogen source. Different carbon sources, including glucose, lactose, soluble starch, maltose, fructose, xylitol, and D-mannitol, were added to replace sucrose as the carbon source. Sucrose was used as the fixed carbon source, and different nitrogen sources, including glycine, urea, beef extract, ammonium sulfate, ammonium chloride, potassium nitrate, and peptone, were used to replace sodium nitrate. A negative control group was set up with no added nitrogen or carbon sources. All treatments were incubated at 25°C in darkness for 7 days with four replicates per treatment.

#### 2.5.3 Effects of temperature on mycelial growth of *Ilyonectria robusta*

The activated strain LJJZ-1 was inoculated onto freshly prepared potato dextrose agar (PDA) plates and incubated at 25°C in a constant-temperature incubator for 7 days until the mycelium fully covered the surface of the plate. Subsequently, an 8 mm mycelial plug was excised using a sterile cork borer, and the plug was inoculated onto the center of a new PDA plate. The inoculated plates were then incubated in constant-temperature incubators set to 4°C, 10°C, 15°C, 20°C, 25°C, 28°C, 30°C, and 40°C, under dark conditions. Four replicates were set for each temperature condition. After 7 days of incubation, the colony diameter was measured using the cross-measurement method.measured after incubation. After 7 days of incubation, the colony diameter was measured using the cross-measurement method.measured after incubation.

#### 2.5.4 Effects of pH on mycelial growth of *Ilyonectria robusta*

Using PDA medium as the base, the pH was adjusted to 4, 5, 6, 7, 8, 9, 10, and 11 by adding 0.1 mol·L^−1^ NaOH or 0.1 mol·L^−1^ HCl solutions. After cooling and solidification, an 8 mm mycelial plug was excised using a sterile cork borer and inoculated at the center of the medium. The plates were incubated at 25°C in a constant-temperature incubator. Four replicates were set for each pH treatment. After 7 days of incubation, colony diameters were measured using the cross-measurement method, and growth status was recorded to evaluate the effects of different pH conditions on strain growth.

#### 2.5.5 Effects of light conditions on mycelial growth of *Ilyonectria robusta*

Using PDA medium as the base, an 8 mm mycelial plug was excised using a sterile cork borer and inoculated at the center of the medium. The plates were incubated under three different light conditions: continuous light for 24 h, continuous darkness for 24 h, and alternating 12 h light/12 h dark cycles. Four replicates were set for each treatment. After 7 days of incubation, colony diameters were measured using the cross-measurement method, and the results were recorded to compare the effects of different light conditions on strain growth.

### 2.6 Screening and evaluation of fungicides for the control of basal stem rot

The mycelial growth rate method was employed to evaluate the inhibitory effects of seven commercially available fungicides on the mycelial growth of *I. robusta*. Detailed information on the fungicides and the concentration gradients is provided in Table 2. Except for 98% technical fludioxonil, which was diluted with dimethyl sulfoxide (DMSO), all fungicides were diluted with sterile distilled water. The diluted solutions were mixed with PDA medium at a ratio of 1:300 (v/v) to prepare the amended media. Fungicide concentrations were selected based on preliminary tests, with specific concentrations listed in Table 2.

**Table 2:** Different chemical fungicides and their concentration gradients.

Fungicides	Amount of active ingredient/mg. L^−1^
Fluazinam 98% TC	0.5, 1.0, 2.0, 5.0, 8.0
Metalaxyl-M·Azoxystrobin 30% SC	20, 50, 100, 150, 200
Metalaxyl·Hymexazol 30% AS	5, 20, 50, 100, 150
Jinggangmycin 24% AS	10, 50, 100, 200, 500
Eugenol 20% EW	50, 100, 150, 200, 250
Berberine 0.5% AS	50, 150, 200, 250, 300
Matrine·Osthol 1.5% AS	100, 200, 300, 400, 500

An 8-mm-diameter mycelial plug was excised from the actively growing margin of the pathogen colony and inoculated onto the center of the fungicide-amended plates. Plates were incubated in darkness at 25°C, with PDA medium without fungicides serving as the control. Each treatment included five replicates. After 7 days of incubation, colony diameters were measured using the cross method, and the inhibition rate was calculated to assess the fungicidal effects against the causal agent of basal stem rot in *S. chinensis*.

The inhibition rate (%) was calculated as follows:


$$\text{PIMG}(\%)=\frac{C-T}{C-F}\times 100\%$$

F refers to the diameter of the fungal plug, C denotes the radial growth diameter of the fungus in the control group, and T indicates the radial growth diameter of the fungus in the treatment group (Usman et al., 2022).

The inhibition rate (y-axis) was plotted against the logarithm of fungicide concentration (log x, x-axis). Linear regression analysis was performed using the least squares method to obtain the toxicity regression equation y = ax + b and the correlation coefficient (r). The effective concentration for 50% inhibition (EC₅₀, mg·L^−1^) was estimated based on the regression equation. Data fitting and statistical analyses were conducted using SPSS 23.0 software (IBM Corp., Armonk, NY, USA).

### 2.7 Data analysis

Experimental data were organized and preliminarily analyzed using Microsoft Excel 2024 (Microsoft Corp., Redmond, WA, USA). Analysis of variance (ANOVA) was performed with SPSS 23.0 (IBM Corp., Armonk, NY, USA), and multiple comparisons were conducted using Duncan’s multiple range test (DMRT) at a significance level of *p* < 0.05. One-way ANOVA was employed to assess the differences in the biological characteristics of the pathogen, while Probit analysis was used to fit the concentration–response relationships and calculate the median effective concentration (EC₅₀). Phylogenetic trees were constructed using MEGA 11.0 (Molecular Evolutionary Genetics Analysis, Pennsylvania State University, USA). Graphs were generated with Origin 2019b (OriginLab Corp., Northampton, MA, USA).

## 3 Results and analysis

### 3.1 Disease symptoms and incidence

**Figure 1:**
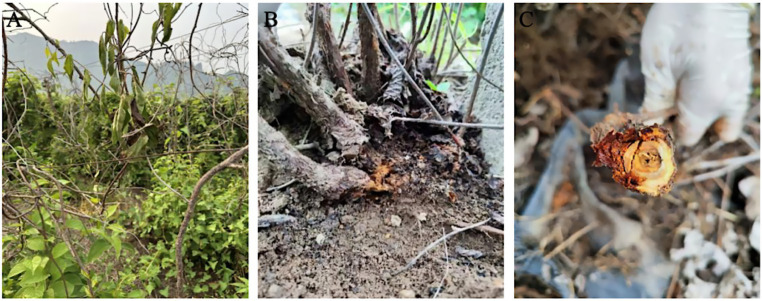
Typical disease symptoms such as basal stem rot, wilting and dieback of *S. chinensis* plants in the field. **(A)** At the beginning of the disease, the leaves started to wilt and turn green. **(B)** Basal stem (about 1–2 cm from the ground) showed browning and rotting. **(C)** In the later stages of the disease, aboveground tissues become necrotic, epidermal rot, and become brittle and breakable.

In July 2024, a field disease survey was conducted in the main production area of *S. chinensis* in Latai Village, Liudougou Town, Linjiang City, Jilin Province. A randomized survey method was used to observe and sample 12 plants showing typical symptoms from representative field plots. The results showed that basal stem rot was widespread in the surveyed fields, with a disease incidence of 100%. Within individual fields, the incidence ranged from 20 to 30%, reaching up to 70% in localized areas. The disease began to appear in early May, gradually developed, and declined by late August, with the peak incidence occurring from July to August. Typical symptoms first appeared at the basal stem 1 to 2 cm above the soil surface, manifesting as dark brown lesions that gradually expanded upward and downward to encircle the stem (Figure 1B). As the disease progressed, leaf wilting and necrosis were observed (Figure 1A). In the later stage, the epidermis of the basal stem decayed, and the pathogen invaded the pith, causing dark brown rot, resulting in brittle stems prone to breakage (Figure 1C).

### 3.2 Isolation of the pathogen and pathogenicity assay

In July 2024, twelve *S. chinensis* samples with basal stem rot were collected from Latai Village, Liudougou Town, Linjiang City, Jilin Province. Tissue blocks were aseptically excised from the epidermis, phloem, xylem, and pith and inoculated onto PDA plates, yielding eleven purified isolates (LJJZ-1 to LJJZ-11). Since all isolates exhibited highly uniform colony morphology on PDA and ITS, *β-tubulin*, and *TEF1-α* sequence comparisons showed no significant differences, the isolate with the lowest designation, LJJZ-1, was selected as the representative strain to avoid redundant experiments and ensure reproducibility. Four inoculation systems—isolated stem segments (8 cm), tissue-cultured seedlings, whole isolated seedlings, and sprouted live seedlings—were then established using the *S. chinensis* cultivar ‘Yeonlip Red’ as the host for pathogenicity assays. The results were as follows:

#### 3.2.1 Detached stem segment inoculation

**Figure 2:**
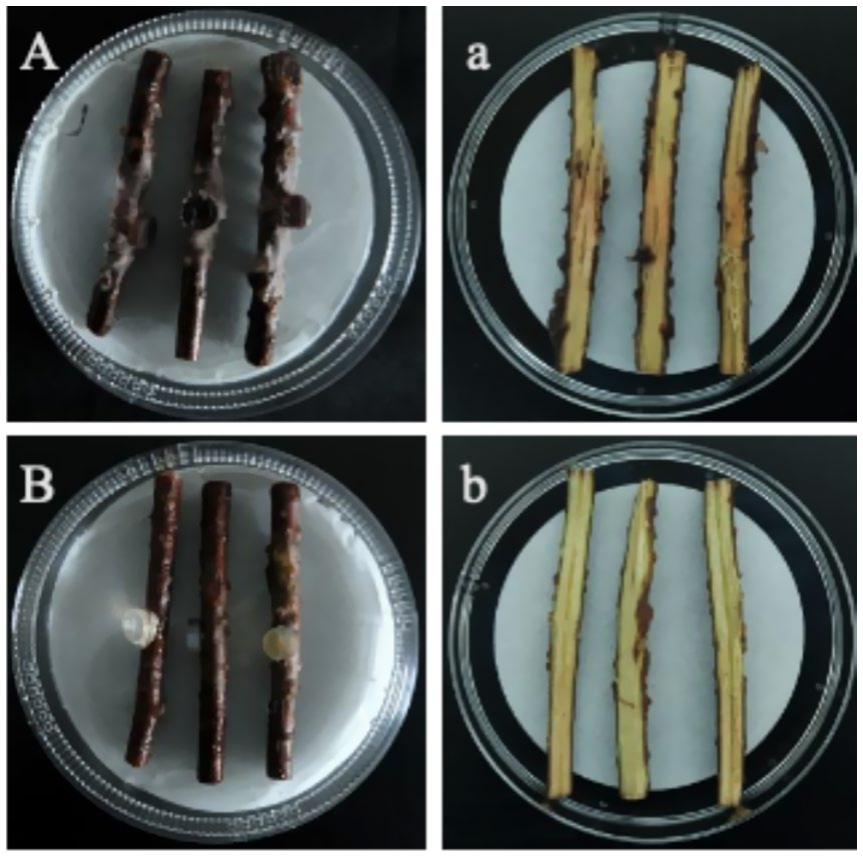
Basal stem rot symptoms 7 days after inoculation. **(A)** Surface of an inoculated stem segment showing abundant fungal mycelia and epidermal decay; **(a)** Corresponding longitudinal section with brown to dark-brown necrotic lesions spreading along the vascular bundles. **(B)** Surface of a non-inoculated control showing no symptoms; **(b)** Corresponding section of the control stem, with no necrosis.

7 days after inoculation, the surfaces of the inoculated stem segments (Figure 2A) exhibited abundant fungal mycelia and epidermal decay, and their corresponding sections (Figure 2a) showed typical basal stem rot symptoms: brown to dark-brown necrotic lesions formed at the inoculation sites and extended both upward and downward along the vascular bundles; no symptoms were observed on the surface (Figure 2B) or in the sections (Figure 2b) of the non-inoculated control segments. Pure cultures with morphological characteristics identical to the original isolate LJJZ-1 were consistently re-isolated from symptomatic tissues in all inoculated treatments, fulfilling Koch’s postulates and confirming LJJZ-1 as the causal agent of basal stem rot in *S. chinensis*.

#### 3.2.2 Inoculation of *in vitro* tissue-culture seedlings

**Figure 3:**
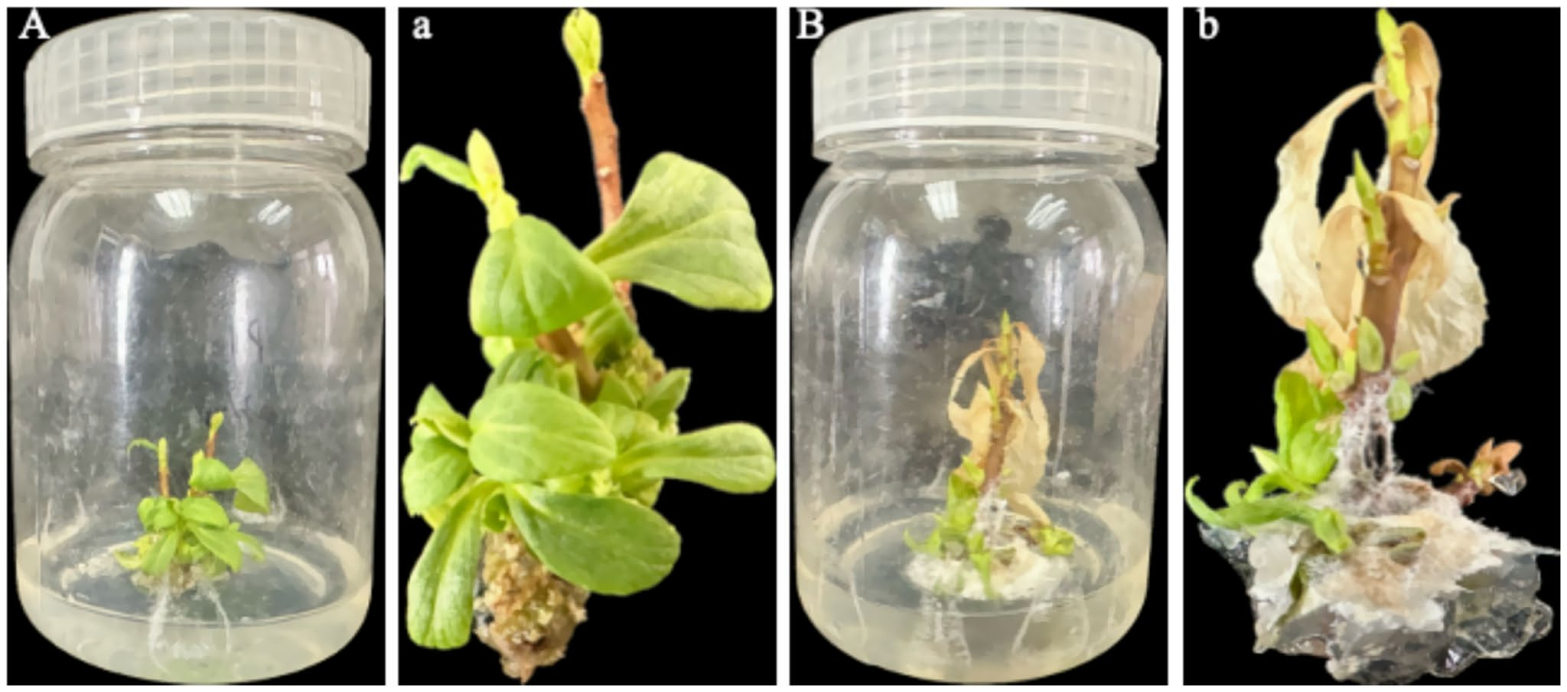
Observation of symptoms and mycelial infection in *in vitro* seedlings 7 d after inoculation. **(A)** Control seedlings in culture vessels showing green leaves with no lesions; **(a)** Upon removal, control seedlings exhibit intact stem bases and leaves without chlorosis or wilting; **(B)** Inoculated seedlings in vessels exhibiting leaf chlorosis and wilting; **(b)** Upon removal, examination of the medium and stem base reveals dark-brown lesions and abundant fungal mycelia.

7 days after inoculation, the control in vitro seedlings within the culture vessels (Figure 3A) displayed green leaves with no infection symptoms; upon removal (Figure 3a), their stem bases remained intact and leaves showed no chlorosis or wilting. In contrast, the inoculated seedlings in the vessels (Figure 3B) exhibited leaf chlorosis and wilting; upon removal, examination of the medium and stem base (Figure 3b) revealed dark-brown lesions and abundant fungal mycelia. Pure cultures with morphological characteristics identical to the original isolate LJJZ-1 were consistently re-isolated from symptomatic tissues in all inoculated treatments, fulfilling Koch’s postulates and confirming LJJZ-1 as the causal agent of basal stem rot in *S. chinensis*.

#### 3.2.3 Inoculation of detached seedlings

**Figure 4:**
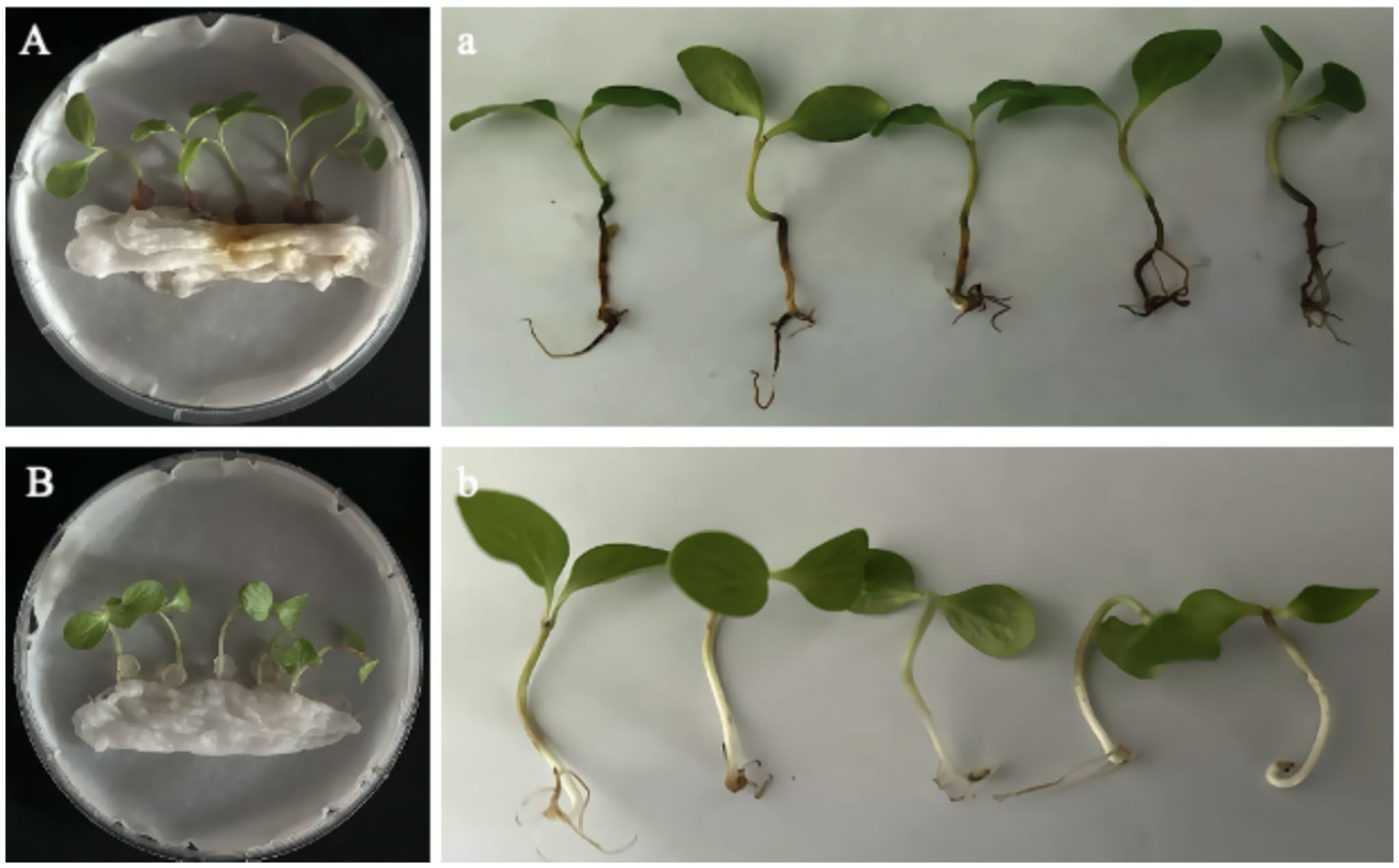
Symptoms of detached seedlings 7 d after inoculation. **(A)** Inoculated seedlings in culture dishes showing dark-brown lesions staining the originally white, cotton-like material at the basal stem; leaves remained green. **(a)** Upon removal, dark-brown rot was observed at the basal stem, extending to the roots. **(B)** Control seedlings in dishes showed no lesion formation or discoloration of the cotton-like material. **(b)** After removal, no browning or decay was observed at the basal stem or roots.

At 7 days post-inoculation, all detached seedlings (*n* = 5) developed rust-colored, ring-shaped lesions on the stem, with tissues becoming brittle and easily broken (Figure 4A); no abnormalities were observed in the stems of control seedlings (Figure 4B). In the culture dishes, the originally white, moist, cotton-like structures at the base of inoculated seedlings were stained dark brown by the lesions, forming distinct signs of infection, while the leaves remained green (Figure 4A). Upon removal, dark-brown rot was observed at the stem base, extending to the roots (Figure 4a). In contrast, control seedlings showed no discoloration or lesions on the cotton-like material in the dishes (Figure 4B), and no browning or decay was detected in the stem base or roots after removal (Figure 4b). Pure cultures with morphological characteristics identical to the original isolate LJJZ-1 were consistently re-isolated from symptomatic tissues in all inoculated treatments, fulfilling Koch’s postulates and confirming LJJZ-1 as the causal agent of basal stem rot in *S. chinensis*.

#### 3.2.4 Inoculation of germinated live seedlings

**Figure 5:**
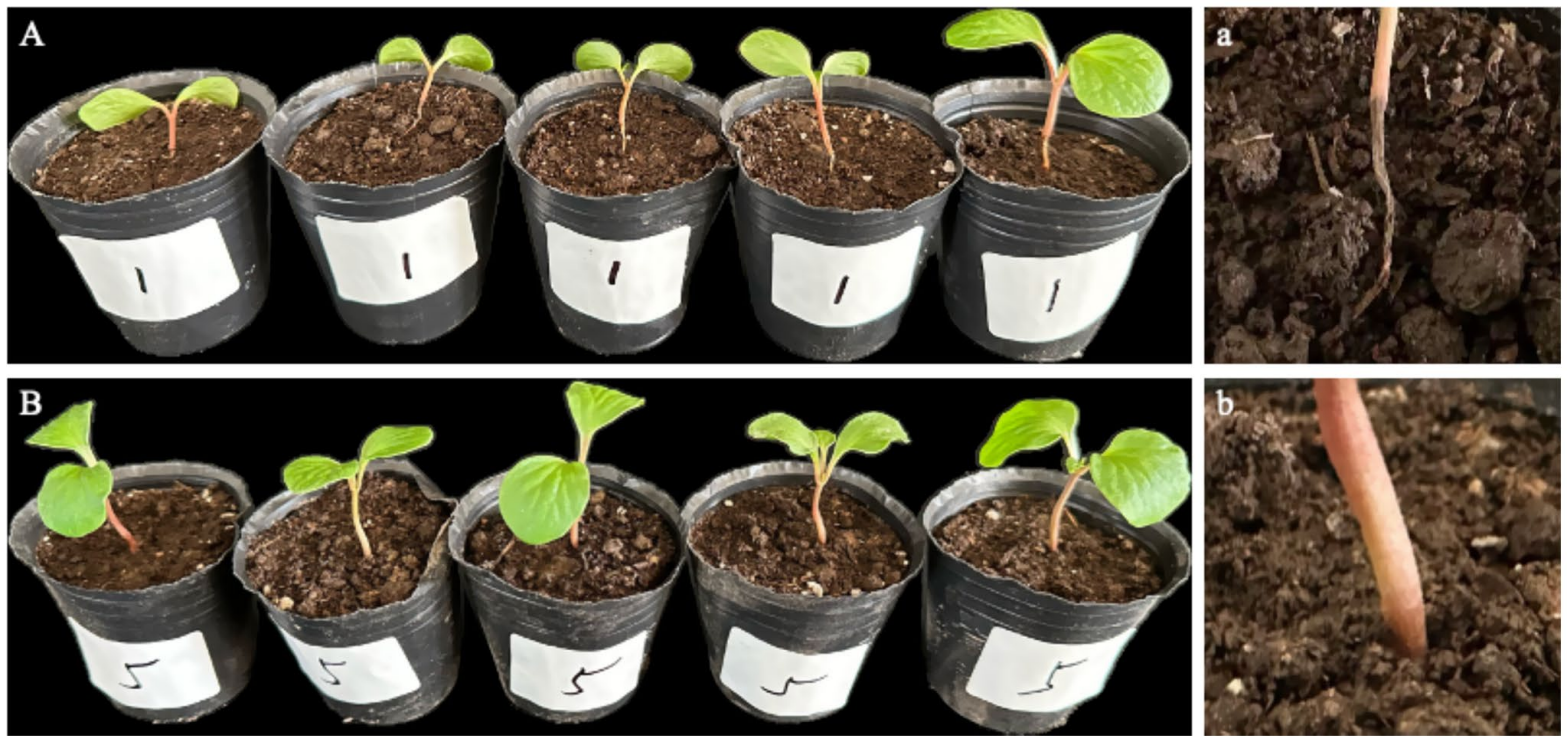
Symptoms of basal stem rot in *S. chinensis* seedlings 14 days after inoculation. **(A)** Typical symptoms showing dark brown lesions encircling the basal stem, with vascular browning, epidermal decay, tissue sloughing, and seedling lodging. **(a)** Representative close-up of the basal stem shrinkage and rot, with leaves remaining green. **(B)** Healthy control seedlings showing no symptoms of rot or discoloration at the basal stem. **(b)** Representative close-up of a control seedling with healthy basal stem and green leaves.

14 days after inoculation, dark brown lesions appeared at the basal stems of live seedlings and gradually extended to encircle the stem. Subsequently, complete browning of the vascular bundles, epidermal decay, tissue sloughing, and noticeable shrinkage were observed, eventually leading to seedling lodging and brittleness (Figure 5A). A typical symptom in Figure 5A is shown in Figure 5a, where the basal stem is visibly shrunken and decayed, while the leaves remain green. In contrast, the control group remained healthy, and no signs of rot or disease were observed at the stem base (Figure 5B). A representative control seedling from Figure 5B is shown in Figure 5b, with a healthy basal stem showing no signs of decay or discoloration, green leaves, and overall vigorous growth. Pathogens re-isolated from all symptomatic tissues of inoculated seedlings consistently produced pure cultures with morphological characteristics identical to those of the original isolate LJJZ-1, fulfilling Koch’s postulates and confirming LJJZ-1 as the causal agent of basal stem rot in *S. chinensis*.

### 3.3 Identification of basal stem rot pathogens of *Schisandra chinensis*

#### 3.3.1 Observation of morphological characteristics

**Figure 6:**
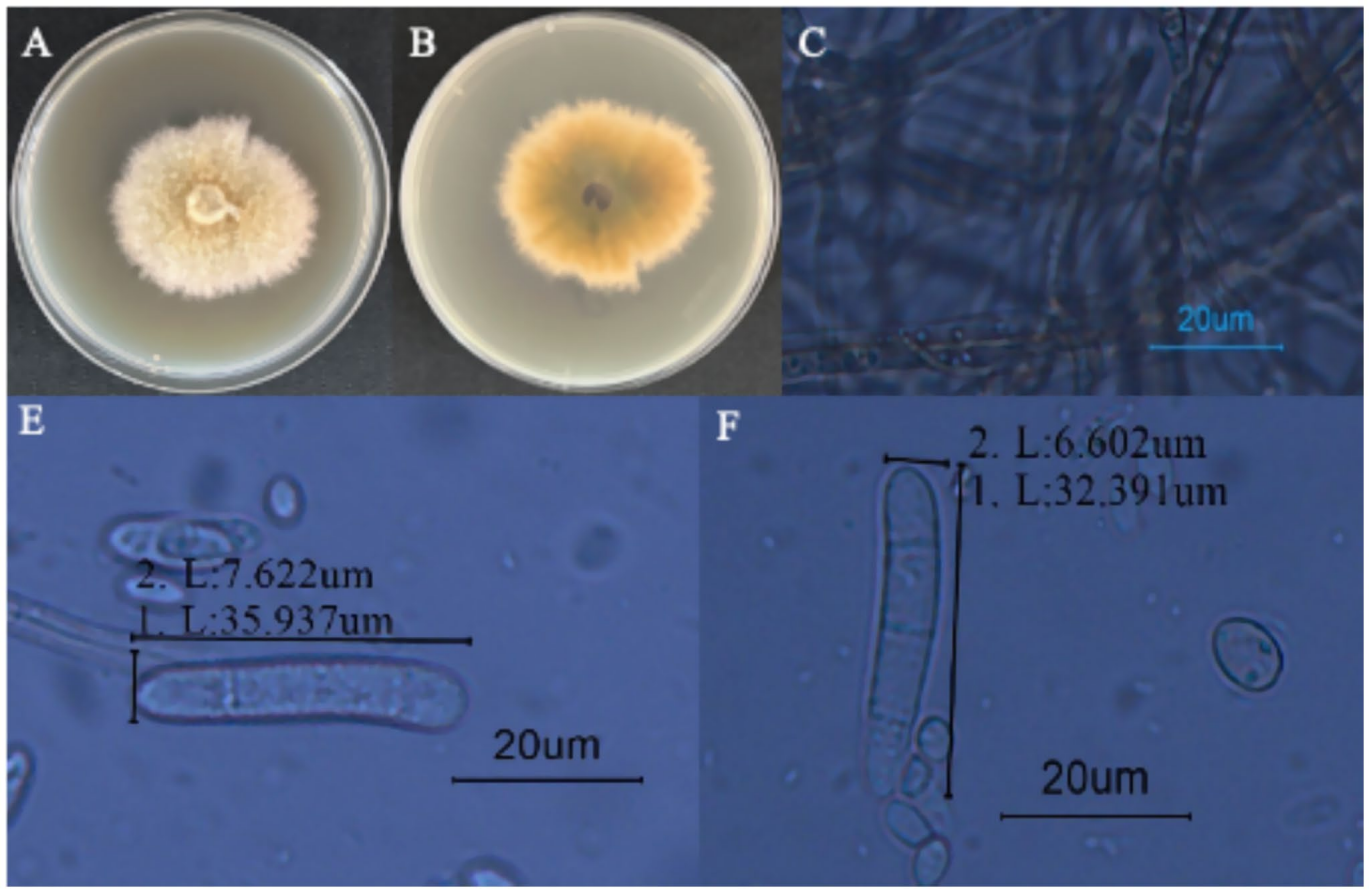
Morphological characteristics of *I. robusta.* **(A)** is the frontal colony morphology of strain LJJZ-1; **(B)** is the colony morphology of strain LJJZ-1; **(C)** is the mycelium morphology of strain LJJZ-1; **(D)** is the large conidium morphology of strain LJJZ-1; **(E, F)** is the small conidium morphology of strain LJJZ-1.

Strain LJJZ-1 was cultured on PDA medium at 25°C for 7 days, forming irregular colonies with slightly regular margins. The colony surface appeared earthy yellow, with a pale yellow periphery and a yellow center; the mycelium was abundant and floccose (Figure 6A). The reverse side of the colony was yellowish brown with patches of earthy yellow (Figure 6B). Microscopic observation revealed that the hyphae were transparent to semi-transparent and clearly septate (Figure 6C). The conidia were cylindrical, straight or slightly curved, with rounded apices and 1–3 septa, occurring singly or in chains (Figures 6D-F); the conidia measured 6.602–7.622 μm in width and 32.391–35.937 μm in length (Figures 6D-F).

#### 3.3.2 Molecular biological characterization

**Figure 7:**
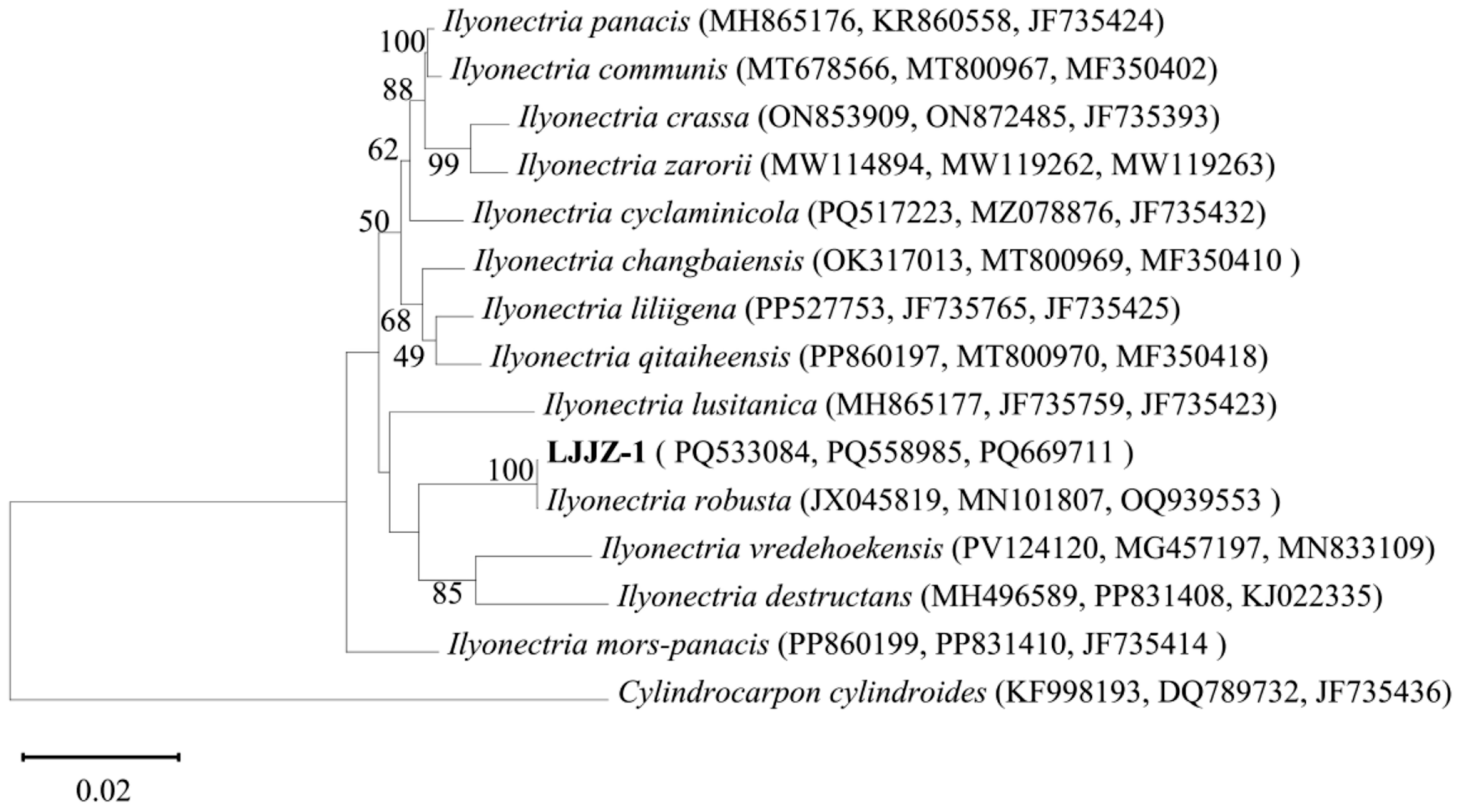
Phylogenetic tree of strain LJJZ-1 and related strains based on sequences of *ITS*, *TUB2* and *TEF1-α* tandem sequences.

The obtained genomic DNA sequences were compared with existing entries in the GenBank database via BLAST, revealing that all three gene fragments of strain LJJZ-1 showed high homology to *I. robusta*: ITS exhibited 100% identity (JX045819), *β*-tubulin 99.73% identity (MN101807), and TEF1-*α* 100% identity (OQ939553). These sequences have been deposited in GenBank under the following accession numbers: ITS (PQ533084), *β-tubulin* (PQ669711), and *TEF1-α* (PQ558985). After sequencing and submission, we concatenated the three gene regions and constructed a Maximum Likelihood phylogenetic tree using MEGA 11.0 (Figure 7). The strains included in this analysis—with details of gene region, species name, strain designation, geographic origin, and GenBank accession number—are listed in Table 3. The phylogenetic tree shows that LJJZ-1 clusters within the same clade as the reference *I. robusta* strains F312a (JX045819), A-19-14 (MN101807), and HYTTL-4 (OQ939553), with a bootstrap support value of 100%, further confirming its phylogenetic affinity. Taken together with morphological characteristics and molecular identification, strain LJJZ-1 is conclusively identified as *I. robusta*.

**Table 3:** *ITS* sequences, *TUB2* and *TEF1-α* gene sequence sources of 14 soil-red husk (*Ilyonectria*) strains.

Gene region	Scientific name	Strain	Geo_loc_name	GeneBank No.
*ITS*	*I. panacis*	CBS 129079	Canada	MH865176
	*I. communis*	H1-9	China	MT678566
	*I. crassa*	NW-FVA1829	Germany	ON853909
	*I. zarorii*	CPC 37837	Chile	MW114894
	*I. cyclaminicola*	Ic	South Korea	PQ517223
	*I. changbaiensis*	YMF 1.07319	China	OK317013
	*I. liliigena*	LK9	China	PP527753
	*I. qitaiheensis*	DR12B	China	PP860197
	*I. lusitanica*	CBS 129080	Portugal	MH865177
	*I. robusta*	F312a	USA	JX045819
	*I. vredehoekensis*	RS4	China	PV124120
	*I. destructans*	17chu02-01	South Korea	MH496589
	*I. mors-panacis*	DR11A	China	PP860199
	*C. cylindroides*	Cyl04	Chile	KF998193
*TEF1-α*	*I. panacis*	320HR12	Austria	KR860558
	*I. communis*	H1-9	China	MT800967
	*I. crassa*	NW-FVA1829	Germany	ON872485
	*I. zarorii*	CPC 37837	Chile	MW119262
	*I. cyclaminicola*	487E	Poland	MZ078876
	*I. changbaiensis*	Q24-5	China	MT800969
	*I. liliigena*	CBS 305.85	Netherlands	JF735765
	*I. qitaiheensis*	R3-2	China	MT800970
	*I. lusitanica*	CBS 129080	Portugal	JF735759
	*I. robusta*	HYTTL-4	China	MN101807
	*I. vredehoekensis*	4,154	China	MG457197
	*I. destructans*	FG191	China	PP831408
	*I. mors-panacis*	FG193	China	PP831410
	*C. cylindroides*	CBS 324.61	Netherlands	DQ789732
*β-Tubulin*	*I. panacis*	CDC-N-9a	Canada	JF735424
	*I. communis*	1,512	China	MF350402
	*I. crassa*	CBS 139.30	Netherlands	JF735393
	*I. zarorii*	CPC 37835	Chile	MW119263
	*I. cyclaminicola*	CBS 302.93	Netherlands	JF735432
	*I. changbaiensis*	4,404	China	MF350410
	*I. liliigena*	CBS 189.49	Netherlands	JF735425
	*I. qitaiheensis*	H309	China	MF350418
	*I. lusitanica*	CBS 129080	Portugal	JF735423
	*I. robusta*	A-19-14	Canada	OQ939553
	*I. vredehoekensis*	4,065	China	MN833109
	*I. destructans*	G. J. S.10–132	USA	KJ022335
	*I. mors-panacis*	CBS 306.35	Canada	JF735414
	*C. cylindroides*	CBS 503.67	Norway	JF735436

### 3.4 Biological characterization of *Schisandra chinensis* basal stem rot pathogen

#### 3.4.1 Effects of different nutrient media on the mycelial growth of *Ilyonectria robusta*

**Figure 8:**
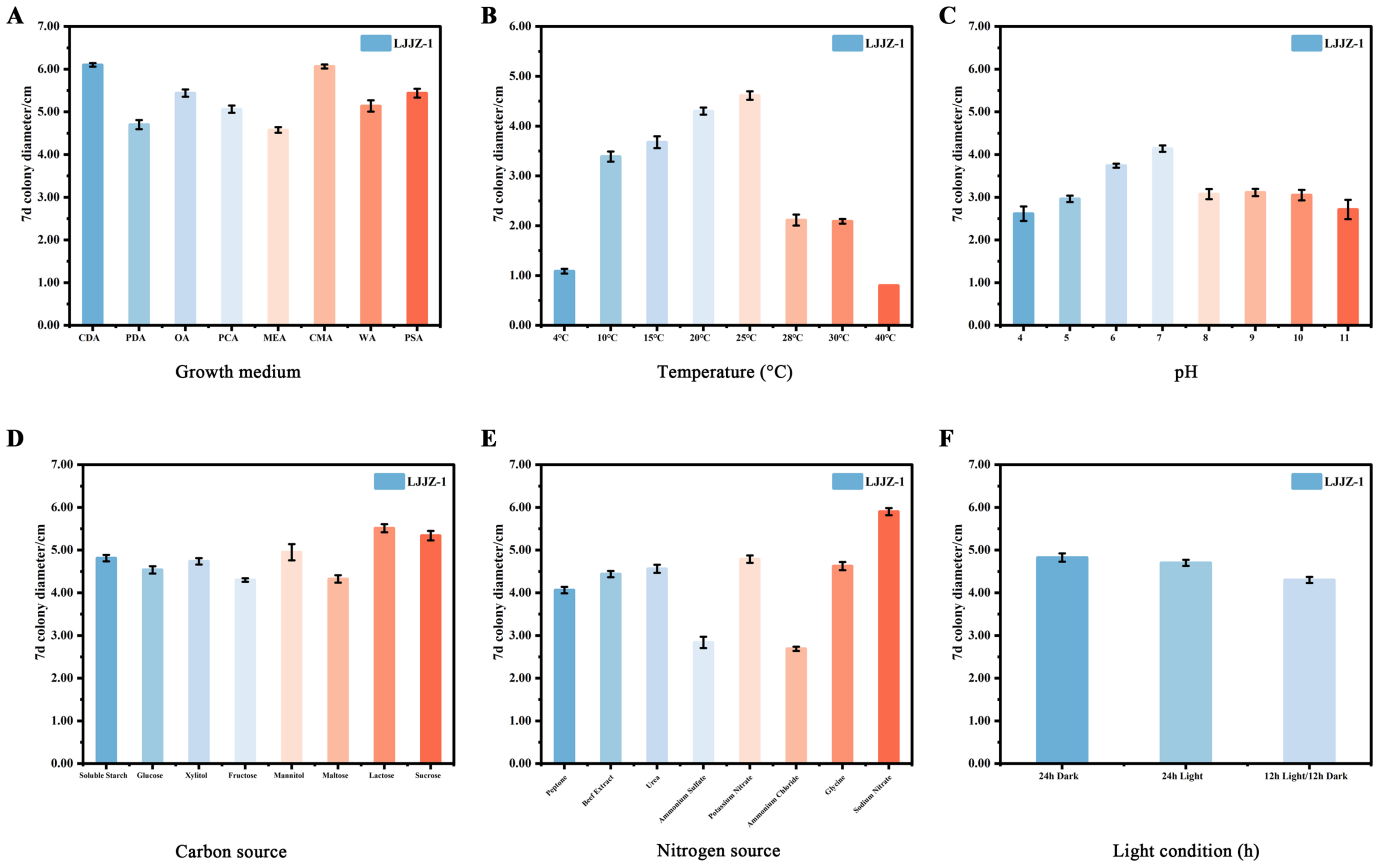
The effects of different media **(A)**, temperatures **(B)**, pH values **(C)**, carbon sources **(D)**, nitrogen sources **(E)**, and light conditions **(F)** on the mycelial growth of *I. robusta*.

As shown in Figure 8A, there were significant differences in the effects of different media formulations on the colony diameter of *I. robusta* (One-way ANOVA, *F* = 412.78, *p* < 0.001). Pathogenic strain LJJZ-1 was able to grow on all eight media tested, with the largest colony diameter in CDA medium (6.10 ± 0.05 cm), which had denser mycelium, comparable to and denser than that of CMA (6.06 ± 0.05 cm), PSA (5.44 ± 0.09 cm), OA (5.44 ± 0.09 cm) and PCA (5.06 ± 0.07 cm) being the next largest, followed by PSA (5.44 ± 0.09 cm), OA (5.44 ± 0.09 cm) and PCA (5.06 ± 0.07 cm), and PCA (5.06 ± 0.07 cm), which was the second largest. 0.07 cm followed by sparser mycelium; MEA medium (4.58 ± 0.06 cm) had the smallest diameter, and PDA (4.70 ± 0.10 cm) was slightly higher than that of MEA. The means and standard deviations of the groups were CDA (6.10 ± 0.05 cm), PDA (4.70 ± 0.10 cm), OA (5.44 ± 0.09 cm), PCA (5.06 ± 0.07 cm), MEA (4.58 ± 0.06 cm), CMA (6.06 ± 0.05 cm), WA (5.14 ± 0.04 cm) and PSA (5.44 ± 0.09 cm). In conclusion, CDA and CMA media were the most favorable for *I. robusta* colony expansion, while peptone and agar substrate formulations represented by MEA were relatively ineffective.

#### 3.4.2 Effects of different temperatures on mycelial growth of *Ilyonectria robusta*

As shown in Figure 8B, different temperature treatments had different effects on the colony diameter of *I. robusta* (One-way ANOVA, *F* = 1232.25, *p* < 0.001). Colony diameter increased significantly with increasing temperature in the range of 4 to 25°C, with a maximum at 25°C (4.61 ± 0.09 cm); when the temperature exceeded 25°C, the colony diameter showed a gradual decreasing trend to a minimum at 40°C (0.80 ± 0.00 cm). The mean and standard deviation of each group were: 4°C (1.09 ± 0.05 cm), 10°C (3.39 ± 0.10 cm), 15°C (3.68 ± 0.12 cm), 20°C (4.30 ± 0.07 cm), 25°C (4.61 ± 0.09 cm), 28°C (2.11 ± 0.11 cm), 30°C (2.09 ± 0.05 cm), and 40°C (0.80 ± 0.00 cm), respectively. Overall, the growth of *I. robusta* was optimal at a moderate temperature of 20 ~ 25°C, while colony expansion was significantly inhibited by both low and high temperatures.

#### 3.4.3 Effects of different pH values on mycelial growth of *Ilyonectria robusta*

As shown in Figure 8C, different pH levels had significant effects on the colony diameter of *I. robusta* [One-way ANOVA, F = (insert *F*-value), *p* < 0.001]. The colony diameter increased with rising pH from 4.0 to 7.0, reaching a maximum at pH 7.0 (4.14 ± 0.08 cm), and then gradually declined under more alkaline conditions. At lower pH (4.0), the average colony diameter was the smallest (2.61 ± 0.17 cm), indicating that acidic environments substantially inhibited mycelial growth. Moderate pH levels (6.0 and 7.0) supported optimal growth, with colony diameters of 3.74 ± 0.05 cm and 4.14 ± 0.08 cm, respectively. A noticeable decline was observed under alkaline conditions, with pH 10.0 and 11.0 yielding diameters of 3.05 ± 0.12 cm and 2.71 ± 0.23 cm, respectively. Among all treatments, the variation in colony diameter was most stable at pH 6.0 (SD = 0.05) and most variable at pH 11.0 (SD = 0.23). Overall, *I. robusta* exhibited optimal mycelial growth at neutral to slightly acidic conditions (pH 6.0–7.0), while both strong acidity and alkalinity negatively affected its colony expansion.

#### 3.4.4 Effects of different carbon and nitrogen sources on the mycelial growth of *Ilyonectria robusta*

As shown in Figure 8D, different carbon source treatments had different effects on the colony diameter of *I. robusta*, a strong soil red crust fungus (One-way ANOVA, *F* = 72.93, *p* < 0.001). Among the eight selected carbon sources, the largest colony diameter was found in lactose medium (5.51 ± 0.09 cm), followed by sucrose (5.34 ± 0.11 cm) and mannitol (4.95 ± 0.19 cm); whereas, fructose (4.30 ± 0.04 cm) and soluble starch (4.81 ± 0.08 cm) were lower, glucose (4.54 ± 0.09 cm), xylitol (4.74 ± 0.08 cm) and maltose (4.33 ± 0.09 cm) were intermediate. Overall, disaccharides (lactose, sucrose) were the most favorable for colony expansion, with monosaccharides and polyols being the next most effective.

As can be seen in Figure 8E, different nitrogen source treatments had different effects on the colony diameter of *I. robusta*, a strong soil red crust fungus (One-way ANOVA, *F* = 563.49, *p* < 0.001). Among the eight selected nitrogen sources, the largest colony diameter was found under sodium nitrate medium (5.90 ± 0.08 cm), followed by urea (4.56 ± 0.09 cm) and beef dip (4.44 ± 0.08 cm); while ammonium chloride (2.69 ± 0.05 cm) and ammonium sulfate (2.84 ± 0.13 cm) were the lowest, and peptone (4.06 ± 0.08 cm), glycine (4.63 ± 0.10 cm) and potassium nitrate (4.79 ± 0.09 cm) were in the middle. Overall, organic nitrogen sources and nitrates were more favorable for *I. robusta* growth, while ammonium salts significantly inhibited colony expansion.

#### 3.4.5 Effects of different pH values on mycelial growth of *Ilyonectria robusta*

As shown in Figure 8F, different light/dark treatments had different effects on the colony diameter of *I. robusta*, a strong soil red crust fungus (One-way ANOVA, *F* = 47.09, *p* < 0.001). The largest colony diameter was observed under 24 h total darkness (4.83 ± 0.10 cm); the second largest under 24 h total light (4.70 ± 0.07 cm); and the smallest under 12 h light/12 h darkness alternating treatment (4.30 ± 0.07 cm). The means and standard deviations of the groups were 24 h dark (4.83 ± 0.10 cm), 24 h light (4.70 ± 0.07 cm) and 12 h light/12 h dark (4.30 ± 0.07 cm), respectively. It is evident that continuous dark conditions were most favorable for *I. robusta* colony expansion, while alternating light and dark significantly inhibited colony growth.

### 3.5 Screening and evaluation of efficacy of agents for the control of basal stem rot caused by *Ilyonectria robusta*

As shown in Table 4, the inhibitory effects of different agents on *I. robusta* showed a significant dose-dependence (logit regression analysis, *p* < 0.001). As shown in Table 4, the inhibitory effects of different fungicides on Ilyonectria robusta showed a significant dose-dependent relationship (logit regression analysis, *p* < 0.001). Among the tested agents, 1.5% Sophora·Osthol exhibited the strongest antifungal activity, with an inhibition rate reaching 100.00% at 500 mg/L (logit 8.72). 30% Metalaxyl-M·Azoxystrobin was the second most effective, achieving 64.99% inhibition at 200 mg/L (logit 5.39). 20% Eugenol also showed strong activity, with an inhibition rate of 61.80% at 250 mg/L (logit 5.30). 98% Fluazinam demonstrated a notable inhibition rate of 54.55% at a very low concentration of 8 mg/L (logit 5.11), indicating a high potency at low doses.In contrast, 0.5% Berberine showed moderate activity, reaching 38.59% inhibition at 300 mg/L (logit 4.71), and 30% Metalaxyl·Hymexazol showed 43.97% inhibition at 150 mg/L (logit 4.85), indicating moderate effectiveness. 24% Validamycin, however, had the weakest inhibitory effect, with only 16.18% inhibition at 500 mg/L (logit 4.01).

Overall, 1.5% Sophora·Osthol demonstrated the highest inhibitory activity against *I. robusta*, followed by 30% Metalaxyl-M·Azoxystrobin and 20% Eugenol. Given its strong efficacy, low toxicity, and broad applicability, Sophora·Osthol can be recommended as the primary candidate fungicide for managing *I. robusta*.

**Table 4:** Inhibition of mycelial growth of *I. robusta* by pharmaceuticals.

Fungicide	Concentration (mg/L)	Log concentration	Colony diameter (cm)	Inhibition rate (%)	Probit value
Control (CK)	–	–	3.87	–	–
30% Metalaxyl·Hymexazol	5	0.6990	3.78	2.9316	3.1091
	20	1.3010	3.38	15.9609	4.0039
	50	1.6990	2.98	28.9902	4.4463
	100	2.0000	2.72	37.4593	4.6803
	150	2.1761	2.52	43.9739	4.8484
Control (CK)	–	–	4.20	–	–
24% Validamycin	10	1.0000	4.03	5.0000	3.3551
	50	1.6990	4.00	5.8824	3.4353
	100	2.0000	3.83	10.8824	3.7672
	200	2.3010	3.75	13.2353	3.8847
	500	2.6990	3.65	16.1765	4.0128
Control (CK)	–	–	4.02	–	–
20% Eugenol	50	1.6990	2.88	35.4037	4.6256
	100	2.0000	2.43	49.3789	4.9844
	150	2.1761	2.37	51.2422	5.0311
	200	2.3010	2.10	59.6273	5.2437
	250	2.3979	2.03	61.8012	5.3002
Control (CK)	–	–	3.78	–	–
0.5% Berberine	50	1.6990	2.98	26.8456	4.3825
	150	2.1761	2.75	34.5638	4.6029
	200	2.3010	2.73	35.2349	4.6210
	250	2.3979	2.70	36.2416	4.6480
	300	2.4771	2.63	38.5906	4.7099
Control (CK)	–	–	4.52	–	–
1.5% Sophora·Osthol	100	2.0000	1.50	81.1828	5.8847
	200	2.3010	1.25	87.9032	6.1702
	300	2.4771	1.05	93.2796	6.4970
	400	2.6021	1.00	94.6237	6.6094
	500	2.6990	0.80	100.0000	8.7190
Control (CK)	–	–	4.17	–	–
30% Metalaxyl-M·Azoxystrobin	20	1.3010	2.77	41.5430	4.7864
	50	1.6990	2.43	51.6320	5.0409
	100	2.0000	2.40	52.52225	5.0633
	150	2.1761	2.28	56.0831	5.1531
	200	2.3010	1.98	64.9852	5.3850
Control (CK)	–	–	3.77	–	–
98% Fluazinam	0.5	−0.3010	3.60	5.7239	3.4203
	1	0.0000	3.33	14.8148	3.9595
	2	0.3010	2.62	38.7205	4.7134
	5	0.6990	2.43	45.1178	4.8774
	8	0.9031	2.15	54.5455	5.1141

A probabilistic-unit regression model was used to determine the virulence of seven agents against *I. robusta*, and their median effective concentrations (EC₅₀; see Table 5) were calculated. EC₅₀ values varied widely from 5.658 to 39078.786 mg/L. 98%Fluazinam proved most potent (EC₅₀ = 5.658 mg/L, *p* < 0.01), followed by 1.5% Sophora·Osthol (EC₅₀ = 36.648 mg/L), 30%Metalaxyl-M·Azoxystrobin (EC₅₀ = 53.122 mg/L), 30%Metalaxyl·Hymexazol (EC₅₀ = 179.920 mg/L), and 20% Eugenol (EC₅₀ = 119.101 mg/L). 0.5% Berberine (EC₅₀ = 1607.413 mg/L) exhibited moderate activity, while 24% Validamycin was least effective (EC₅₀ = 39078.786 mg/L). In terms of model fit, 0.5% Berberine (*R*^2^ = 0.981) and 1.5% Sophora·Osthol (R^2^ = 0.975) exhibited excellent linearity, whereas 24% Validamycin (*R*^2^ = 0.903) showed a relatively poorer fit, mainly because high concentrations precipitate in the medium, preventing the use of higher gradients. Regarding mechanisms of action, 98% Fluazinam achieves nanoscale inhibition by disrupting the mitochondrial respiratory chain; 30% Metalaxyl·Hymexazol interferes with fungal RNA synthesis, arresting pathogen growth; 20% Eugenol disrupts cell membrane integrity, causing cytoplasmic leakage; 0.5% Berberine, derived from Coptis chinensis, is naturally low in toxicity and readily biodegradable; 1.5% Sophora·Osthol—a complex multi-component formulation—offers multi-target synergistic potential; 30% Metalaxyl-M·Azoxystrobin inhibits cytochrome-c oxidase, blocking energy metabolism; and 24% Validamycin inhibits fungal *α*-amylase activity.

**Table 5:** Indoor virulence determination of seven agents against strain *I. robusta.*

Agent	Toxicity regression equation	Coefficient of determination (*R*^2^)	EC_50_ (mg/L)
30% Metalaxyl·Hymexazol	y = 1.8953x − 4.2741	*R*^2^ = 0.968	179.920
24% Validamycin	y = 0.8528x − 3.9162	*R*^2^ = 0.903	39078.786
20% Eugenol	y = 1.5220x − 3.1595	*R*^2^ = 0.970	119.101
0.5% Berberine	y = 0.6583x − 2.1107	*R*^2^ = 0.981	1607.413
1.5% Sophora·Osthol	y = 3.0644x − 4.7929	*R*^2^ = 0.975	36.648
30% Metalaxyl-M·Azoxystrobin	y = 0.7994x − 1.3792	*R*^2^ = 0.875	53.122
98% Fluazinam	y = 2.1355x-1.6074	*R*^2^ = 0.919	5.658

## 4 Discussion

This study is the first to report *I. robusta* as the causal pathogen of basal stem rot disease of *S. chinensis* in Northeast China, confirming its occurrence in Linjiang City, Jilin Province, the main production area of *S. chinensis*. As a traditional medicinal plant with important value, the cultivation area of *S. chinensis* has been continuously expanding. However, soilborne diseases have increasingly become severe in recent years, seriously restricting the sustainable development of the industry. Although disease symptoms are frequently observed in the field, systematic studies on the pathogens remain insufficient. This study identified *I. robusta* (strain LJJZ-1) as the dominant pathogen causing this disease, which is significantly different from previously reported root rot or stem rot pathogens dominated by Fusarium species in Liaoning Province (Xue et al., 2007). Unlike the common root rot diseases in other regions, the disease caused by *I. robusta* mainly concentrates on the basal stem segment 1–2 cm above the root-stem junction, initially showing browning and necrosis, spreading along the vascular bundles, ultimately leading to plant wilting and death. Notably, the root system of affected plants remains largely intact, suggesting that the infection pathway and pathogenic mechanisms may differ from typical root pathogens. Field surveys conducted in 2024 showed that the disease is widespread in some orchards in Jilin Province, with individual plots exhibiting incidence rates exceeding 30% and disease severity reaching 100%, indicating a severe control challenge. Phylogenetic analysis based on ITS, *TEF-1α*, and *β-tubulin* gene sequences confirmed the pathogen as *I. robusta*, which is clearly distinct from *Fusarium* pathogens previously reported. *I. robusta* is a broad-host-range pathogen that has been confirmed to infect wheat (Gao et al., 2023), Zea mays (Wang et al., 2025), Panax ginseng (Zhang et al., 2025), Panax quinquefolius (Shao et al., 2022), astragalus (Wang et al., 2024), Astragalus membranaceus (Wang et al., 2024), and Corchorus olitorius (Tang et al., 2022), indicating high potential for spread in crop rotation or intercropping systems. The physiological and biochemical characteristics of strain LJJZ-1 demonstrated high adaptability to the summer climatic conditions of Jilin Province, with optimal growth temperature between 20 and 25°C and pH 6.0–7.0. The colony grows rapidly under high humidity. These conditions align well with the average summer temperature (22–25°C), relative humidity (65–80%), and local soil characteristics (brown soil, pH 5.5–6.5, high organic matter content) in Jilin, indicating good ecological adaptability of the pathogen to the local environment. Among eight culture media tested, the fastest colony growth occurred on CDA and CMA media (6.10 ± 0.05 cm and 6.06 ± 0.05 cm, respectively), with dense mycelia. The physicochemical properties of CDA medium are similar to Jilin brown soil, suggesting that the nutrient environment highly matches the ecological niche of the pathogen. In addition, lactose (5.51 ± 0.09 cm) and sodium nitrate (5.90 ± 0.08 cm) were the most suitable carbon and nitrogen sources, respectively, indicating that organic-rich soils with nitrate nitrogen fertilization may promote pathogen survival and expansion. Conversely, ammonium nitrogen sources such as ammonium chloride and ammonium sulfate significantly inhibited growth, suggesting that nitrogen source regulation could be a strategy for disease management. Light treatment experiments showed that LJJZ-1 grew best under continuous darkness (4.83 ± 0.10 cm), followed by continuous light (4.70 ± 0.07 cm), and least under alternating 12 h light/12 h dark conditions (4.30 ± 0.07 cm). This is consistent with the semi-shaded understory environment of *S. chinensis* cultivation, indicating that current shading management may unintentionally create a microenvironment conducive to pathogen spread, warranting re-evaluation of light management in disease control. Overall, *I. robusta* is widely present in *S. chinensis* production areas in Jilin and is highly adapted to local soil, climate, and cultivation systems. It can rapidly expand in high humidity, organic-rich, poorly aerated environments and possesses long-term survival capability. The pathogen may persist in soil residues, rhizosphere fragments, and microaggregates, forming an “inoculum corridor” that facilitates cross-infection in long-term monoculture and multiple crop systems, making traditional crop rotation insufficient to completely interrupt the pathogen source. Currently, there are no specific fungicides available on the market for controlling basal stem rot disease of *S. chinensis*. *In vitro* fungicide screening results showed that 98% Fluazinam exhibited the best efficacy (EC₅₀ = 5.658 mg/L), followed by 1.5% Sophora·Osthol and 30% Metalaxyl-M·Azoxystrobin, while biological pesticides such as 24% Validamycin showed poor effects, indicating the need to develop targeted control products. Given the pathogen’s strong adaptability and covert spread, integrated management strategies are recommended. We suggest systematic control from the following aspects: (1) Establish a qPCR-based pathogen monitoring system for precise early warning; (2) Optimize crop rotation to avoid consecutive planting with highly susceptible hosts such as ginseng; (3) Improve soil structure and increase beneficial microbial inputs; (4)Strengthen field sanitation by timely removal of diseased residues and soil treatment.

Future research should focus on large-scale pathogen surveys and elucidating the pathogenic mechanisms on *S. chinensis*. Notably, pathogen detection rates were significantly higher in plots with ginseng as the previous crop, suggesting pathogen accumulation associated with crop history, forming a “disease complex system.” Multi-omics approaches (metagenomics, transcriptomics) to dissect pathogen-host interactions may provide theoretical support for breeding disease-resistant varieties and developing novel biocontrol strategies, laying a foundation for sustainable development of the *S. chinensis* industry in Northeast China.

## 5 Conclusion

Basal stem rot is a common and destructive soil-borne disease in the cultivation of *S. chinensis*, often leading to collar rot, leaf yellowing, and even plant death, which seriously affects yield and fruit quality and poses a potential threat to industry development. Due to the pathogen’s cryptic nature and complex transmission pathways, identifying the causal agent and understanding its biological characteristics are essential for developing effective and science-based control strategies. Therefore, this study conducted the first systematic investigation of basal stem rot in *S. chinensis* in Jilin Province, focusing on pathogen identification, biological characterization, and fungicide screening. The main findings are as follows: The causal agent was identified as *I. robusta* based on morphological characteristics and multilocus sequence analysis (ITS, *β-tubulin*, *TEF1-α*). Pathogenicity was confirmed using four different inoculation materials, fulfilling Koch’s postulates. I*n vitro* characterization showed that the pathogen grows optimally at neutral pH, 25°C, and high humidity—conditions typical of summers in northeastern China—and exhibits maximal colony development on Czapek–Dox agar (CDA) and potato dextrose agar (PDA) under continuous darkness, with lactose and sodium nitrate preferred as carbon and nitrogen sources, respectively. Among seven fungicides tested *in vitro*, 98% Fluazinam demonstrated the strongest inhibition (EC₅₀ = 5.658 mg·L^−1^), followed by 1.5% matrine–serpentine (EC₅₀ = 36.648 mg·L^−1^) and 30% pyrimethanil–fludioxonil (EC₅₀ = 179.920 mg·L^−1^); 24% jinggangmycin and other biopolypeptide agents showed limited efficacy. Given the pathogen’s cryptic, soilborne nature, integrated management is recommended, including removal of infected plants, crop rotation, soil amendment, and optimized planting density. Fields previously cultivated with ginseng require thorough disinfection and strategic rotation before establishing *S. chinensis*. Because *I. robusta* is highly adaptable to diverse hosts—including ginseng, soybean, and maize—and can persist in soil or crop residues, there is a significant risk of cross-infection to neighboring crops. Therefore, monitoring of adjacent fields, avoidance of continuous planting with susceptible species, and establishment of buffer zones are critical to limiting pathogen spread.

This work not only identifies the pathogen and its key environmental preferences driving basal stem rot in Jilin Province but also lays the groundwork for rapid diagnosis, chemical control, and resistance breeding. Future research should expand field surveys across a broader area and employ omics approaches to elucidate infection mechanisms, thereby guiding the development of durable, sustainable disease-management strategies.

## Data Availability

The authors acknowledge that the data presented in this study must be deposited and made publicly available in an acceptable repository, prior to publication. Frontiers cannot accept a manuscript that does not adhere to our open data policies.
